# Conformational epitopes of myelin oligodendrocyte glycoprotein are targets of potentially pathogenic antibody responses in multiple sclerosis

**DOI:** 10.1186/1742-2094-8-161

**Published:** 2011-11-17

**Authors:** Til Menge, Patrice H Lalive, H -Christian von Büdingen, Claude P Genain

**Affiliations:** 1 Neuroimmunology Laboratories, Department of Neurology, University of California San Francisco, CA, USA; 2 Department of Neurology, Medical Faculty, Heinrich-Heine-University, Düsseldorf, Germany; 3 Department of Neurosciences, Division of Neurology, Faculty of Medicine, University of Geneva, Switzerland and Department of Pathology and Immunology, Faculty of Medicine, University of Geneva, Switzerland

**Keywords:** Antibodies, Autoimmunity, Multiple sclerosis, Myelin, Biomarkers

## Abstract

**Background:**

Myelin/oligodendrocyte glycoprotein (MOG) is a putative autoantigen in multiple sclerosis (MS). Establishing the pathological relevance and validity of anti-MOG antibodies as biomarkers has yielded conflicting reports mainly due to different MOG isoforms used in different studies. Because epitope specificity may be a key factor determining anti-MOG reactivity we aimed at identifying *a priori *immunodominant MOG epitopes by monoclonal antibodies (mAbs) and at assessing clinical relevance of these epitopes in MS.

**Methods:**

Sera of 325 MS patients, 69 patients with clinically isolated syndrome and 164 healthy controls were assayed by quantitative, high-throughput ELISA for reactivity to 3 different MOG isoforms, and quantitative titers correlated with clinical characteristics. mAbs defined unique immunodominant epitopes distinct to each of the isoforms.

**Results:**

In the majority of human samples anti-MOG levels were skewed towards low titers. However, in 8.2% of samples high-titer anti-MOG antibodies were identified. In contrast to anti-MOG reactivity observed in a mouse model of MS, in patients with MS these never reacted with ubiquitously exposed epitopes. Moreover, in patients with relapsing-remitting MS high-titer anti-MOG IgG correlated with disability (EDSS; Spearman r = 0.574; p = 0.025).

**Conclusions:**

Thus high-titer reactivity likely represents high-affinity antibodies against pathologically relevant MOG epitopes, that are only present in a small proportion of patients with MS. Our study provides valuable information about requirements of anti-MOG reactivity for being regarded as a prognostic biomarker in a subtype of MS.

## Introduction

Autoantibodies directed against myelin antigens have been a long-standing focus of interest in multiple sclerosis (MS) research, especially those binding to myelin oligodendrocyte glycoprotein (MOG). MOG is predominantly expressed in the CNS, and is exposed on the outermost lamellae of the myelin sheath thus readily available for a humoral immune attack [1]. MOG induces demyelinating experimental allergic encephalomyelitis (EAE), the animal model of MS, in a variety of species both by active immunization and by passively transferred anti-MOG antibodies (reviewed by [1,2]). Only those anti-MOG antibodies directed against conformational epitopes, as opposed to linear epitopes, appear to be pathogenic in EAE [3-5]. Recently, it was shown that the murine monoclonal antibody (mAb) 8.18.c5 specific for rat MOG, that confers demyelination, maps to a discontinuous epitope of the surface exposed FG loop of rat MOG, that is also exposed on murine and human MOG [6].

To date, the measurement of serum anti-MOG antibodies using various techniques and different MOG preparations has resulted in inconsistent results and limited reproducibility (reviewed by [7,8]). This study was thus designed to assess the MOG epitope usage in humans employing a novel quantitative high-throughput ELISA. The serum anti-MOG antibody responses of 325 patients with MS, 69 patients with a first demyelinating event (clinically isolated syndrome, CIS) and 164 healthy controls (HC) were assayed. Three isoforms of recombinant MOG were generated and the differential exposure of immunodominant epitopes characterized by a panel of monoclonal anti-MOG antibodies. Restricted patterns of anti-MOG reactivity could be observed in samples with sustained anti-MOG reactivity at high serum dilutions, defining high-titer reactivity. In this cohort we find that anti-MOG antibody levels strongly correlate with disease severity.

## Materials and methods

### Antigens and Antibodies

Three recombinant human MOG isoforms were used for the study. The first, spanning the extracellular domain, amino acids 1-125, (rhMOG_125_) was expressed and purified under physiological conditions as described previously [9]. Secondly, a seven amino acids shorter rhMOG protein, spanning the amino acids 1-118 (rhMOG_118_) was created by usage of a different 3'-end primer: 5'-ATCCATGAGATCTAGGATCTTCTACTTTCAATTCCATTGCTGCC-3', and was expressed and purified as above. Finally, recombinant rat MOG, amino acids 1-125 (ratMOG_125_) was produced in E. coli and purified as described previously [10]. Purity was confirmed to be > 95% by SDS-PAGE (additional file 1) and correct folding ascertained by circular dichroism (additional file 1) [11,12].

The murine monoclonal IgG 8.18C5 against native rat MOG was a gift of Dr. Chris Linington [13]. The marmoset Fab-fragments (Fabs) designated M26, M3-24, and M3-8 derived from a ratMOG_125_-immune animal were generated in our laboratory as described previously [10].

### Patients

325 MS patients meeting the diagnostic criteria for clinically definite MS [14,15], and 69 patients with a first demyelinating were recruited for this study [15]. 36% of the MS patients were treated with either interferon beta or glatiramer acetate at the time of sampling. Patients treated with glucocorticoids within three months or on immunosuppressive therapy within six months of phlebotomy were excluded. 164 volunteers served as healthy controls (HC). Informed consent was obtained from all subjects, and the study was conducted in accordance with Institutional Review Board approval. The clinical characteristics of the patients and HC are summarized in the table contained in additional file 2. Blood was drawn by venipuncture and clotted serum stored at -40°C.

### High-throughput ELISA

Optimal protein concentrations for coating were determined in preliminary experiments; 0.5 μg of rhMOG_125 _and rhMOG_118 _and 1.0 μg of ratMOG_125 _were coated in PBS overnight on a single 384-well microtiter assay plate (Maxisorb, Nunc, Rochester, NY). Control wells were coated with 1.0 μg BSA. To quantify the antibody reactivity, an IgG standard curve was created by coating human IgG (I4506, Sigma, St. Louis, MO) in two-fold dilutions. After washing, plates were blocked for 2 hours with 1% BSA in PBS supplemented with 0.05% Tween 20. Then, human sera were added at three dilutions starting at 1/200, and incubated for 90 min. A positive control known to be reactive to all three rMOG isoforms, and a negative control omitting serum were included. Bound antibodies were detected by an alkaline phosphatase-labeled anti-human IgG (A9544, Sigma) and the optical density (OD) read at 405 nm wavelength in a microplate reader (SpectraMax, Molecular Devices, Sunnyvale, CA) after 30 minute incubation with para-nitrophenyl phosphate (Moss, Pasadena, MD). All samples were tested coded in a blinded fashion in duplicates, standard curves in quadruplicates; incubation was at room temperature (RT), except coating which was at 4°C. Sample handling and ELISA procedures were performed by a robotic workstation (Biomek FX, Beckman Coulter, Fullerton, CA). MOG-ELISA for the monoclonal reagents (8.18c5 and Fab fragments) were performed in 96-well Maxisorb plates according to the protocol as outlined above. 8.18c5 was detected by an anti-mouse IgG (A9044, Sigma) and the Fabs by Protein L (Pierce). Both were peroxidase labeled and developed by 3,3',5,5'-tetramethylbenzidine (Pierce) and the OD read at 450 nm wavelength.

### Denaturing anti-MOG ELISA

To further demonstrate, that conformational MOG-epitopes are conserved in regular ELISA assays as described above, we compared binding of anti-MOG antibodies to recombinant MOG antigens coated under native (previous section) and denaturing conditions. The rMOG isoforms were coated as outlined above in 96-well plates. After washing coated MOG was denatured by incubation with 8 M urea supplemented with 10 mM dithiothreitol (DTT) for 4 hours at RT followed by addition of 25 mM 2-iodoacetamide (IDAA) for fixation. After overnight incubation at RT and extensive washing wells were blocked as above, subjected to either 8.18c5 or anti-His mAb (Invitrogen, Carlsbad, CA), and binding was detected as above.

### Data processing and statistical analysis

The ODs were corrected for the individual background binding and the amount of specific IgG bound to the well interpolated from the on-plate standard curve. Additionally, results were expressed as the signal-to-background *binding ratio *(BR), calculated as the ratio of OD signal/background where applicable, as were results for 8.18c5 and the Fabs.

For the assessment of high-titer reactivity, samples above to 95^th ^percentile of IgG concentrations were tested in a separate experiment in serial dilutions, those with BRs ≥ 2 at 1/3,200 dilution (i.e. signals against MOG that were at least two-fold above the BSA signal) were identified and their mean BR at 1/800 dilution defined as the cut-off for high-titer reactivity. This procedure was done for all three MOG isoforms. This procedure is one of the rigorous methods accepted for defining subtype cut-offs with stringent criteria.

Statistical analysis was conducted using Prism 4.0 (GraphPad, San Diego, CA). Categorical variables were compared using the χ^2^-test, continuous variables using ANOVA and ordinal as well as not normally distributed continuous variables by Kruskal-Wallis testing. The Student-Newman-Keuls method and Dunn's test were used to determine differences in between groups.

## Results

### Demonstration of conformationally coated MOG in ELISA

**Figure 1:**
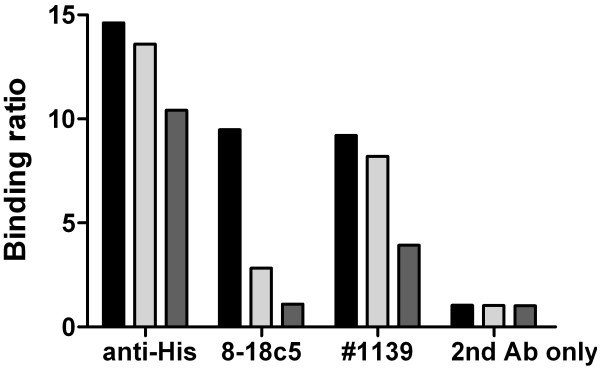
**Denaturing ELISA**. Comparison of rhMOG_118 _coated under physiological conditions in PBS (black bars), after partial denaturing of rhMOG_118 _by incubation with 8 M urea (light grey bars) and after irreversibly completely denaturing by incubation with 8 M urea, 10 mM DTT, 25 mM IDAA (dark grey bars). Results are expressed as binding ratios as described in the methods section. Anti-His denotes a mAb specific for 6-mer histidine peptide tag; #1139 is a human serum serving as positive control in the quantitative ELISA.

Prior to assessing the putative immunodominant MOG epitopes it was shown that coating of MOG to the ELISA plate by electrostatic forces did not denature the protein. Making use of the mAb 8.18c5 that is known to exclusively bind to conformational, but not linear MOG epitopes [5,6], recombinant MOG was denatured by urea and DTT after coating, and the linearized polypeptide fixated by the alkylating sulfhydryl reagent IDAA that binds covalently with cysteine and thus prevents renaturing of MOG. While 8.18c5 reactivity is lost after denaturing/fixating, anti-His mAb reactivity is sustained indicating the MOG polypeptide remains coated in the wells (exemplified for rhMOG_118_; Figure 1).

Thus, coating does not denature MOG to an extent that pathogenic conformational epitopes are destroyed or become unexposed. This however, does not prevent antibodies reactive to linear epitopes to bind to coated MOG as exemplified for human sample #1139 (Figure 1) and as shown previously [3,9]. Because at least half of the reactivity of serum #1139 is lost after denaturing of rhMOG_118 _the samples contains both antibodies against linear epitopes and those exclusively reactive to conformational epitopes (Figure 1).

### Definition of distinct epitopes exposed on the different MOG isoforms

**Figure 2:**
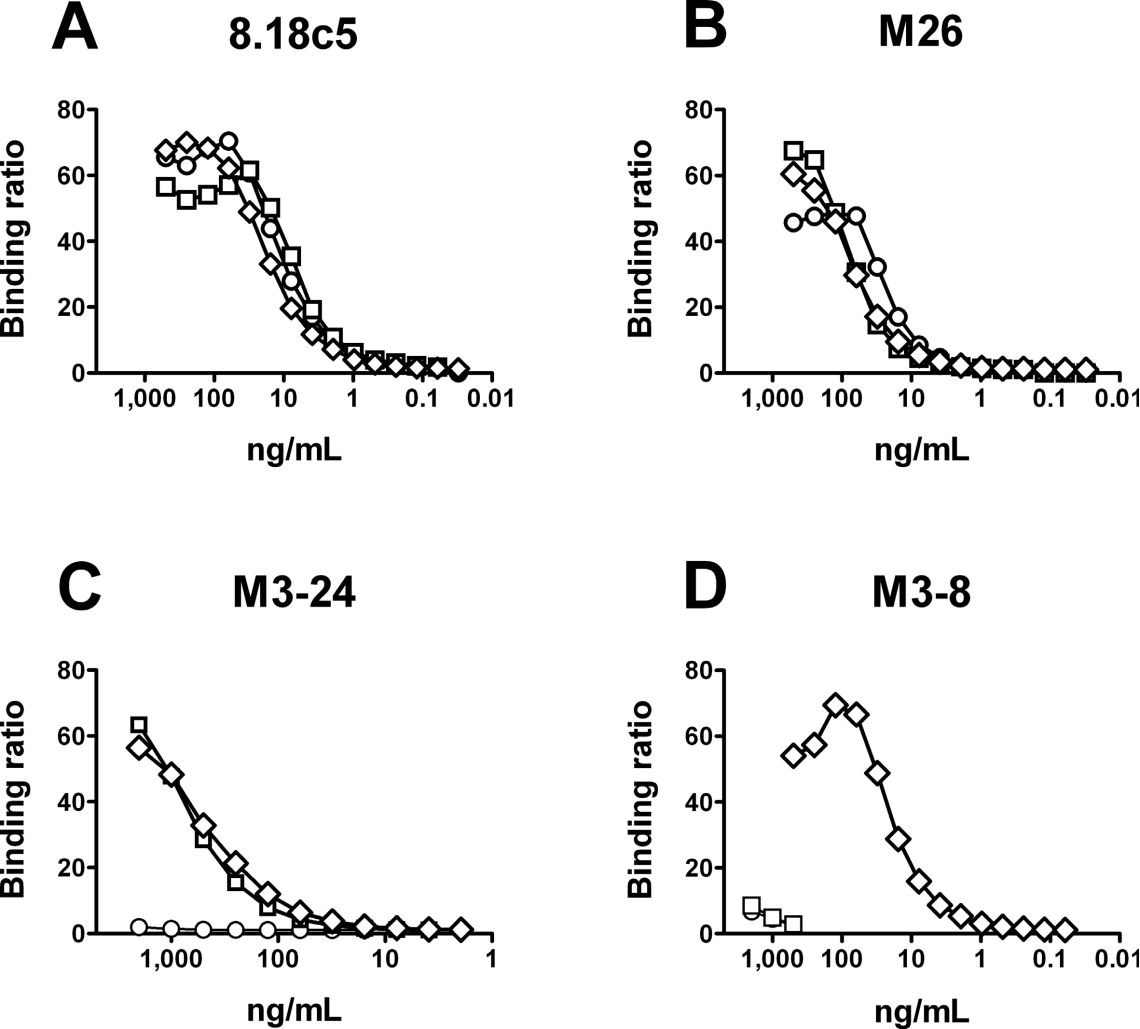
**Monoclonal reagents define distinct epitopes on MOG**. Serial two-fold dilution series of the mouse monoclonal antibody 8.18c5 (A), and the marmoset-derived Fab fragments M26 (B), M3-24 (C) and M3-8 (D) against rhMOG_118 _(-○-), rhMOG_125 _(-□-) and ratMOG_125 _(-◊-).

Despite 90% sequence homology between rhMOG_125 _and ratMOG_125_, and a mere 7 amino acid difference in length between rhMOG_118 _and rhMOG_125_, each of the antigenic isoforms used in this ELISA displayed unique immunodominant epitopes of MOG or combinations thereof, as demonstrated by the mAb 8.18c5 and the monoclonal Fabs M26, M3-24 and M3-8:

- 8.18c5 and M26 bound equally well to all isoforms, defining epitopes commonly exposed on all three MOG isoforms (Figure 2A+B).

- A second epitope, defined by the marmoset Fab M3-24, is unique to rhMOG_125 _and ratMOG_125_, and is not exposed on rhMOG_118_; hence this epitope is species-independent, but dependent on the length of the protein (Figure 2C).

- Thirdly, the Fab M3-8 recognizes an epitope uniquely exposed on ratMOG_125_, but on neither of the human proteins; hence this epitope is species-dependent, (Figure 2D).

It has been previously shown that these Fabs do not inhibit each other's binding or binding of 8.18c5, nor do they recognize linear MOG-derived peptides, which corroborates our interpretation that the different MOG isoforms expose distinct, conformation dependent, immunodominant epitopes [10]. Thus, the use of these three different recombinant isoforms affords to measure antibodies against specific conformational epitopes of MOG in serum.

### Anti-MOG reactivity in healthy controls and cohorts of patients with MS

**Figure 3:**
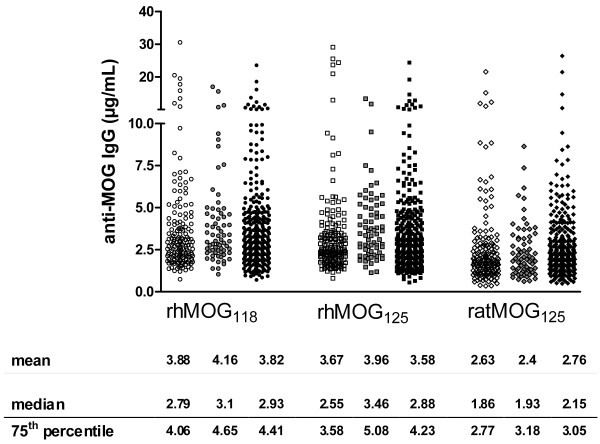
**Scatter plot of ELISA reactivity of healthy controls and patients with CIS and MS against rhMOG_118_, rhMOG_125 _and ratMOG_125_**. Differences between 164 healthy controls (open symbols), 69 CIS patients (grey symbols) and 325 MS patients (solid symbols) for the three MOG preparations, rhMOG_118 _(-○-), rhMOG_125 _(-□-) and ratMOG_125 _(-◊-). Results expressed as IgG concentrations in μg/mL serum.

The quantitative results for antibodies against the 3 different MOG isoforms in 325 patients with MS, 69 CIS patients and 164 HC samples obtained by high-throughput ELISA are summarized in Figure 3. Anti-MOG IgG concentrations below 5.0 μg/mL were over-represented, resulting in a skewed distribution (75^th ^percentile < 5.1 μg/mL for all groups; Figure 3). Anti-rhMOG_125 _reactivity was significantly elevated in the CIS patient group compared to MS and HC (p < 0.05), and anti-ratMOG_125 _significantly higher in MS compared to HC (p < 0.05); however, extensive overlap between groups was observed and the clinical relevance of this finding remains unclear (Figure 3).

Importantly, age, gender, disease duration, or treatment could be excluded as confounding factors in any of the MS subgroups or HC (data not shown).

### Identification and fine epitope specificity of subjects with high-titer anti-MOG IgG

Because of the skewed distribution in favor of low serum concentration antibodies, we sought to identify high-titer, higher affinity samples reasoning that these would be immunologically relevant antibodies reflecting a true immune response, and investigated their reactivity patterns. The quantitative assay with multiple serial dilutions is capable of providing this information in two ways; first, it allows to measure actual antibody concentrations; second, it allows differentiating antibodies that retain reactivity at high dilutions, which may be indicative of higher affinity and could not otherwise be measured in a complex mixture of serum IgG. To specifically identify samples with high-titer reactivity and to differentiate those from the ones that show reactivity only at lower dilutions, we defined cut-offs for BR for the 1/800 dilution, derived from samples with anti-MOG IgG concentrations above the 95^th ^percentile and with sustained antibody reactivity at 1/3,200 dilution (see Methods; additional file 3). Applying these cut-offs, 8.2% of all HC, CIS and MS samples were identified as high-titer reactive; differences between groups were not statistically significant (table 1).

**Table 1:** Differential reactivity against the three different MOG isoforms in samples with high-titer reactivity

	Percentage of samples tested (proportion)^a^	Monospecific (1 antigen only)				Oligospecific (2 antigens)				All 3 antigens
		%	rhMOG_118 _(n)	rhMOG_125 _(n)	ratMOG_125 _(n)	%	rhMOG_118_ + rhMOG_125 _(n)	rhMOG_118_ + ratMOG_125 _(n)	rhMOG_125_ + ratMOG_125 _(n)	**rhMOG_118_**, rhMOG_125_, ratMOG_125_ % (n)
**HC**	10.4 (17/169)	47.1	5	1	2	47.1	7	1	0	5.9 (1)
**CIS**	7.3 (5/69)	80.0	3	1	0	20.0	1	0	0	0
**MS**	6.8 (22/325)	68.2	9	6	0	31.8	5	2	0	0
	
**RR-MS**	7.8 (15/192)	66.7	6	4	0	33.3	4	1	0	0
**SP/PP-MS**	5.3 (7/133)	71.4	3	2	0	28.6	1	1	0	0

Percentage of samples with identified high-titer reactivity to have high-titer reactivity exclusively to one MOG isoform, two of the MOG isoforms used or all three MOG isoforms, respectively. There are no significant differences between HC, CIS and MS or any of the MS subgroups (p > 0.05, χ^2 ^test). Samples were deemed high-titer reactive, if the respective BR at the 1/800 serum dilution was above the defined cut-off BRs (5.8, 5.6, 9.4 for rhMOG_118_, rhMOG_125 _and ratMOG_125_).

**^a ^**refer to additional file 2 for characteristics of all samples tested.

Detailed analysis of the high-titer sera revealed that the majority of these reacted with only one of the human MOG isoforms, either rhMOG_118 _or rhMOG_125 _("monospecific"), or with both of them ("oligospecific", table 1). Concomitant reactivity to all three MOG antigens was only found in one HC sample (table 1). This pattern was consistent in all groups, and there were no differences between early disease (CIS) and chronic forms of MS (SPMS, PPMS). This dichotomy of reactivity is corroborated by the observation that anti-rhMOG_118 _IgG concentrations correlated well with the respective anti-rhMOG_125 _IgG concentrations in the oligospecific high-titer samples (Spearman r = 0.574; p = 0.025), but not in the monospecific high-titer samples (Spearman r = -0.176; p = 0.4; data not shown).

These results underscore the findings of unique epitope exposure as defined by the mAbs (Figure 2) and may be indicative of three major immunodominant epitopes relevant to the human anti-MOG response: [1] one epitope exclusively exposed on rhMOG_118_, [2] one exclusively exposed on rhMOG_125_, and [3] finally one shared epitope exposed on both rhMOG_118 _and rhMOG_125_. None of these seem to be sufficiently exposed on ratMOG_125_. Overall, the anti-MOG reactivity in humans with high-titer antibody responses appears to be diverse and not exclusively directed against epitopes that are commonly exposed (as defined by the mAb 8.18c5 or the Fab M26).

### Clinical correlations of anti-MOG IgG

**Figure 4:**
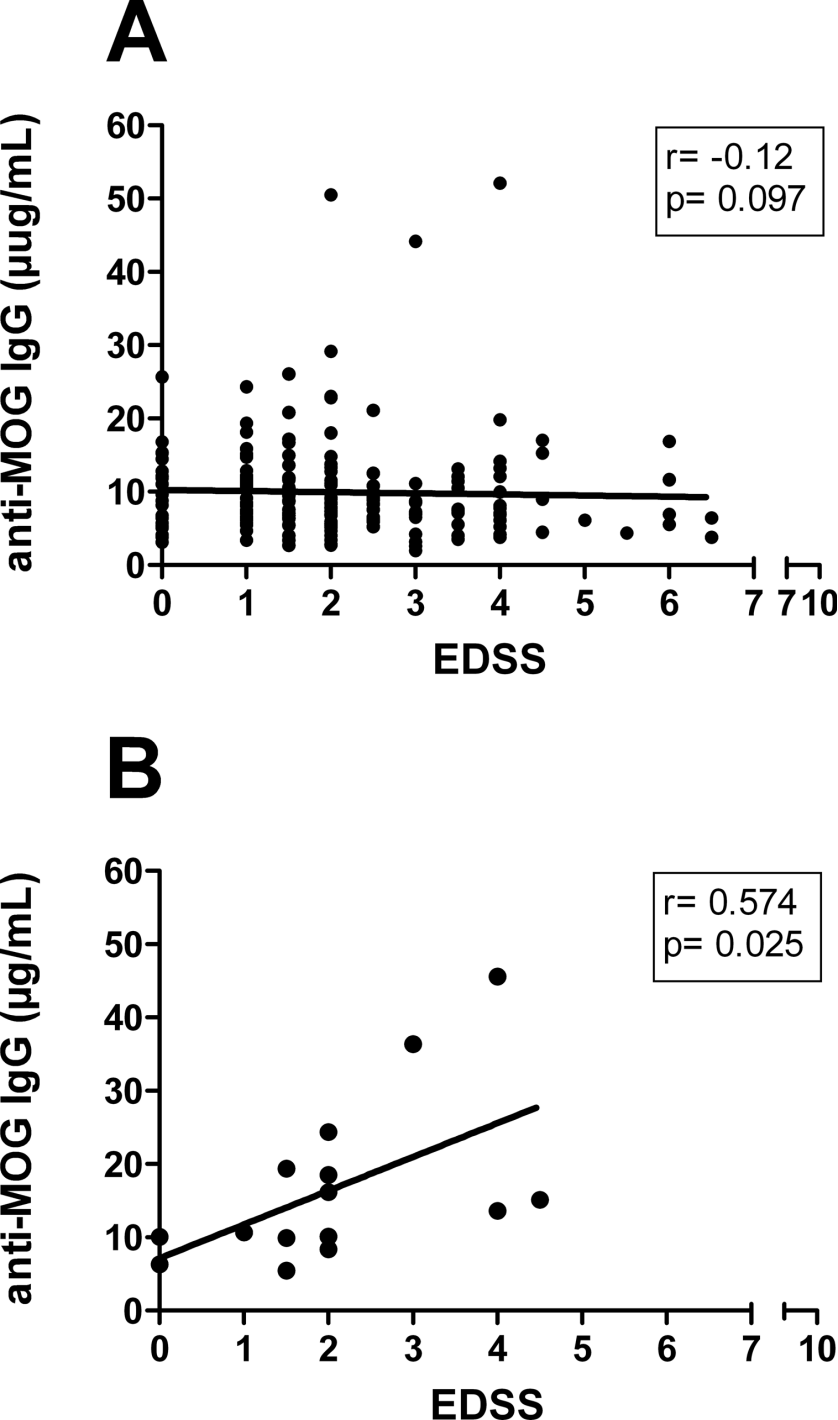
**Correlation of anti-MOG IgG concentration to disease disability in high-titer RR-MS samples**. Lack of correlation of the magnitude of combined reactivity against rhMOG_118_, rhMOG_125 _and ratMOG_125 _(expressed as anti-MOG IgG concentration) with EDSS in all 192 RR-MS patients tested (A); positive correlation in the subset of RR-MS patients with high-titer anti-MOG reactivity with EDSS (B): Spearman r = 0.574; p = 0.025.

In neither the entire MS cohort, nor in any of the subtypes (RR-, SP-, PP-MS individually) were anti-MOG antibody titers correlated with the degree of disability as measured by the extended disability status scale (EDSS) (depicted for RR-MS in Figure 4A) when considering the whole cohorts. In contrast, when isolating the subtype of RR-MS samples with high-titer reactivity, their cumulative anti-MOG IgG concentrations, i.e. the sum of the respective high-titer anti-MOG IgG concentrations (rhMOG_118_, rhMOG_125_, ratMOG_125_, where applicable), positively correlated with the EDSS at blood sampling (Spearman r = 0.574; p = 0.025; Figure 4B). There was no correlation observed for samples of MS patients with chronic disease. Neither age, gender nor disease duration were identified as confounding factors for the EDSS correlation.

For six RR-MS patients longitudinal blood samples were analyzed in a single ELISA assay and demonstrated no significant variation in anti-MOG antibody titers over time (additional file 4). This indicates that pending exceptions, these titers are quite stable over time, excluding titer variability at blood sampling (for example, elevated total IgG due to infection) and that few blood sample measurements would be necessary to define the level of anti-MOG antibody reactivity.

## Discussion

Numerous studies have attempted to assess the presence and clinical implications of serum antibodies against MOG in MS reporting a wide range for the prevalence of anti-MOG antibodies (0% - 78%) (reviewed in [7,8]). The reason for this variability are likely differences in patient populations, assay techniques (e.g., Western blotting, ELISA, RIA), and MOG antigen preparations used [7,16]. Furthermore, an important factor to be taken into consideration is the biophysical environment of MOG in the respective assay system. In particular, denaturing conditions may render proteins linearized and thus inaccessible to pathogenic antibodies targeting conformation-dependent epitopes.

The approach presented here addresses two critical criteria: the diversity of the conformational MOG epitope repertoire at the molecular level, and implementation of a fully quantitative assay of the MOG-directed antibody response. In a preliminary experiment we demonstrated that in our ELISA assay antigen coating does not denature MOG (Figure 1), but rather harsh chemical forces would be necessary to fully linearize MOG rendering it inaccessible for antibodies specific for conformational MOG epitopes. We are thus confident that potentially pathogenic conformational anti-MOG antibodies can be detected by our ELISA. We are therefore able to stratify our analyses according to parameters of potential pathological relevance, an essential step towards meaningful interpretation of serum antibodies. We and others have previously shown that single target antigen ELISA may not sufficiently discriminate between pathogenic anti-MOG antibodies, naturally occurring low-affinity autoantibodies or non-pathogenic antibodies directed against linear MOG epitopes [3,9,17]. To further emphasize the importance of antibody epitope characterization, we have recently reported in CIS patients a high prevalence of antibodies against native, membrane-embedded MOG that do not fully cross-react with recombinant MOG preparations as demonstrated by cytometry and competition assays [18].

The most relevant finding of this study was derived from 8.2% of samples that were identified as high-titer reactive. In these samples, we set out to further characterize the diversity of the human serum anti-MOG antibody repertoire with respect to binding to conformational epitopes that was otherwise masked in the entire cohort predominantly consisting of low-titer anti-MOG reactive antibodies (table 1 Figure 3). At least three immunodominant epitopes became apparent, distinctly exposed on three different recombinant MOG "isoforms". These isoforms display only minor differences in terms of amino acid sequence (table 1) and have no other purpose than to rigorously define specific epitopes. These findings of distinct epitope exposure were corroborated using a panel of differentially reactive anti-MOG monoclonal agents (Figure 2) [10]. Importantly, we present data (Figure 1) and have previously shown that relevant conformational MOG epitopes are preserved in ELISA systems [3,10]. Only after isolating high-titer samples, a strong association of circulating anti-MOG IgG with disability in RR-MS patients could be unmasked (Figure 4). This was achieved by combining the antibody reactivity against all identified putative target epitopes. RatMOG_125 _was included in our panel of MOG antigens in order to assess the reactivity against an ubiquitously exposed common epitope, and because of a previously demonstrated cross-reactivity between ratMOG_125 _and human anti-MOG IgG [10]. The proportion of high-titer samples appears low at first sight; it is however, quite consistent with recent findings of the frequencies of MOG-reactive T cells in the peripheral blood of MS patients, that ranged between approximately 2-4 percent of T cells [19,20]. Interestingly and complementing our data, the frequency of these specific T cells correlated to disease activity and disability [19].

It was an unexpected finding that reactivity to epitopes conserved between species, such as those defined by 8.18c5 or M26 was not predominant in MS high-titer samples (Figure 2A+B, table 1). Rather, specific reactivity appeared to be exclusively directed against restricted epitopes of human MOG (table 1). In contrast, humoral responses in rodent EAE are predominantly directed against the 8.18c5 defined epitope [6].

This diversity in epitope recognition may explain why previous studies employing different MOG preparations have generated such divergent results [7,16]. Indeed, only one previous study has reported a comparable clinical correlation in 262 patients with MS of which 14% were deemed anti-MOG positive by comparison to the HC samples [21]. Most other studies have tested considerably smaller numbers of MS patients, and may have not achieved sufficient statistical power to reveal correlations with clinical parameters. To put in perspective, in Type I diabetes mellitus, large sample numbers of 1,300 to over 4,000 per study were needed to identify the prognostic value of autoantibodies in offspring of diabetic patients, with less than 5% of samples showing high-titer reactivity against the respective antigens [22].

We are aware, that our study does not sufficiently address the issue of specificity that has lacked in previous assays too [7,8], as exemplified by similar overall frequencies of high-titer reactivity in HC compared to MS and CIS (Figure 3; table 1). However, it is striking, that high-titer anti-MOG reactivity in HC includes epitopes of rodent MOG, while that in MS and CIS patients does not. While our study was clearly not designed to prove pathogenicity of anti-MOG antibodies as shown previously by passive transfer experiments in animals [3,23], our findings do suggest, that potentially pathogenic anti-MOG antibody responses are highly specific for certain epitopes of human MOG. In this context it was recently shown that demyelination is augmented in an animal model of virally induced mild demyelination if the animals are engineered to produce anti-MOG IgG prior to infection [24]. Thus, it is intriguing to speculate that anti-MOG antibodies in HC render the same pathogenic potential as in MS patients, but HC individuals have not encountered the pathogenetically relevant viral infection. Furthermore, we cannot rule out the possibility, that the high-titer anti-MOG response occurs secondary to acquired myelin destruction and may as such reflect the magnitude of clinical disability [25].

In summary, we demonstrate for the first time that target epitopes of autoantibody responses are differentially exposed and that the immune response in humans is restricted to distinct epitopes but not identical in all patients and certainly not identical to rodent EAE. Only in a fraction of samples a strong and sustained anti-MOG response can be detected, deemed high-titer reactivity. We report that in RR-MS patients with such reactivity the amount of anti-MOG antibodies are correlated with disability. Our findings bear significant clinical relevance that may have been overlooked in previous studies due to smaller sample sizes. This study supports the concept that establishing certain serum autoantibodies as predictive biomarkers in MS may be possible.

## Abbreviations

ANOVA: analysis of variance; BR: binding ratio; BSA: bovine serum albumin; CD: circular dichroism; CIS: clinically isolated syndrome; CNS: central nervous system; EAE: experimental allergic encephalomyelitis; EDSS: expanded disability status scale; HC: healthy control; HT: high-titer; mAb: monoclonal antibody; MOG: myelin oligodendrocyte glycoprotein; rMOG: recombinant rat MOG (extracellular domain); MS: multiple sclerosis; OD: optical density; PBS: phosphate buffered saline; PP-MS: primary progressive MS; RR-MS: relapsing remitting MS; SNK: student Newman-Keuls test; SP-MS: secondary progressive MS.

## Competing interests

The authors declare that they have no competing interests.

## Authors' contributions

TM designed the study, performed the experiments, analyzed data, wrote the paper. PHL analyzed data, wrote the paper and performed experiments. H-CvB performed experiments, wrote the paper and reviewed the manuscript. CPG wrote the paper and reviewed the manuscript. TM and CPG had full access to all of the data in the study and take responsibility for the integrity of the data and the accuracy of the data analysis. All authors read and approved the final manuscript.

## Supplementary Material

Additonal file 1 **SDS-PAGE and CD-spectroscopy of MOG**. Figures of a SDS-PAGE demonstrating high purity of the three recombinant MOG isoforms, and of a circular dichroism spectroscopy experiment proving correct β-sheet folding. Click here for file

Additonal file 2 **Clinical characteristics of healthy control, CIS and MS samples tested**. Table compiling the demographic and clinical features of the three patient groups. Click here for file

Additonal file 3 **Dilution series of samples with high titers**. Figure of an ELISA of two-fold serial serum dilutions against the three MOG isoforms to demonstrate sustained ELISA reactivity of samples with high IgG concentrations (> 95^th ^percentile) beyond dilutions of 1/2,000. Click here for file

Additonal file 4 **Persistent anti-MOG reactivity in serial samples**. Figure of an ELISA reactivity against rhMOG_118 _of six samples, for which longitudinal samples were drawn every three months over 18 months; the data prove that anti-MOG IgG concentrations vary only within the limits of the assay. Click here for file

## References

[B1] Johns TG Bernard CC The structure and function of myelin oligodendrocyte glycoprotein J Neurochem 1999 72 1 9 9886048 10.1046/j.1471-4159.1999.0720001.x

[B2] von Büdingen HC Tanuma N Villoslada P Ouallet JC Hauser SL Genain CP Immune responses against the myelin/oligodendrocyte glycoprotein in experimental autoimmune demyelination J Clin Immunol 2001 21 155 170 10.1023/A:1011031014433 11403222

[B3] von Büdingen HC Hauser SL Ouallet JC Tanuma N Menge T Genain CP Frontline: Epitope recognition on the myelin/oligodendrocyte glycoprotein differentially influences disease phenotype and antibody effector functions in autoimmune demyelination Eur J Immunol 2004 34 2072 2083 10.1002/eji.200425050 15259004

[B4] Mathey E Breithaupt C Schubart AS Linington C Commentary: Sorting the wheat from the chaff: identifying demyelinating components of the myelin oligodendrocyte glycoprotein (MOG)-specific autoantibody repertoire Eur J Immunol 2004 34 2065 2071 10.1002/eji.200425291 15259003

[B5] Breithaupt C Schubart A Zander H Skerra A Huber R Linington C Jacob U Structural insights into the antigenicity of myelin oligodendrocyte glycoprotein Proc Natl Acad Sci USA 2003 100 9446 9451 10.1073/pnas.1133443100 12874380 PMC170938

[B6] Breithaupt C Schafer B Pellkofer H Huber R Linington C Jacob U Demyelinating myelin oligodendrocyte glycoprotein-specific autoantibody response is focused on one dominant conformational epitope region in rodents J Immunol 2008 181 1255 1263 18606679 10.4049/jimmunol.181.2.1255

[B7] Reindl M Khalil M Berger T Antibodies as biological markers for pathophysiological processes in MS J Neuroimmunol 2006 180 50 62 10.1016/j.jneuroim.2006.06.028 16934337

[B8] Bielekova B Martin R Development of biomarkers in multiple sclerosis Brain 2004 127 1463 1478 10.1093/brain/awh176 15180926

[B9] Menge T von Büdingen HC Lalive PH Genain CP Relevant antibody subsets against MOG recognize conformational epitopes exclusively exposed in solid-phase ELISA Eur J Immunol 2007 37 3229 3239 10.1002/eji.200737249 17918203

[B10] von Büdingen HC Hauser SL Fuhrmann A Nabavi CB Lee JI Genain CP Molecular characterization of antibody specificities against myelin/oligodendrocyte glycoprotein in autoimmune demyelination Proc Natl Acad Sci USA 2002 99 8207 8212 10.1073/pnas.122092499 12060766 PMC123046

[B11] O'Connor KC Appel H Bregoli L Call ME Catz I Chan JA Moore NH Warren KG Wong SJ Hafler DA Wucherpfennig KW Antibodies from inflamed central nervous system tissue recognize myelin oligodendrocyte glycoprotein J Immunol 2005 175 1974 1982 16034142 10.4049/jimmunol.175.3.1974 PMC4515951

[B12] Gori F Mulinacci B Massai L Avolio C Caragnano M Peroni E Lori S Chelli M Papini AM Rovero P Lolli F IgG and IgM antibodies to the refolded MOG(1-125) extracellular domain in humans J Neuroimmunol 2011 233 216 220 10.1016/j.jneuroim.2010.11.011 21215463

[B13] Linnington C Webb M Woodhams PL A novel myelin-associated glycoprotein defined by a mouse monoclonal antibody J Neuroimmunol 1984 6 387 396 10.1016/0165-5728(84)90064-X 6207204

[B14] Poser CM Paty DW Scheinberg L McDonald WI Davis FA Ebers GC Johnson KP Sibley WA Silberberg DH Tourtellotte WW New diagnostic criteria for multiple sclerosis: guidelines for research protocols Ann Neurol 1983 13 227 231 10.1002/ana.410130302 6847134

[B15] McDonald WI Compston A Edan G Goodkin D Hartung HP Lublin FD McFarland HF Paty DW Polman CH Reingold SC Sandberg-Wollheim M Sibley W Thompson A van den Noort S Weinshenker BY Wolinsky JS Recommended diagnostic criteria for multiple sclerosis: guidelines from the International Panel on the diagnosis of multiple sclerosis Ann Neurol 2001 50 121 127 10.1002/ana.1032 11456302

[B16] Amor S Giovannoni G Antibodies to myelin oligodendrocyte glycoprotein as a biomarker in multiple sclerosis--are we there yet? Mult Scler 2007 13 1083 1085 10.1177/1352458507084431 17967836

[B17] Marta CB Oliver AR Sweet RA Pfeiffer SE Ruddle NH Pathogenic myelin oligodendrocyte glycoprotein antibodies recognize glycosylated epitopes and perturb oligodendrocyte physiology Proc Natl Acad Sci USA 2005 102 13992 13997 10.1073/pnas.0504979102 16172404 PMC1236555

[B18] Lalive PH Menge T Delarasse C Della GB Pham-Dinh D Villoslada P von Budingen HC Genain CP Antibodies to native myelin oligodendrocyte glycoprotein are serologic markers of early inflammation in multiple sclerosis Proc Natl Acad Sci USA 2006 103 2280 2285 10.1073/pnas.0510672103 16461459 PMC1413723

[B19] Bahbouhi B Pettre S Berthelot L Garcia A Elong NA Degauque N Michel L Wiertlewski S Lefrere F Meyniel C Delcroix C Brouard S Laplaud DA Soulillou JP T cell recognition of self-antigen presenting cells by protein transfer assay reveals a high frequency of anti-myelin T cells in multiple sclerosis Brain 2010 133 1622 1636 10.1093/brain/awq074 20435630

[B20] Raddassi K Kent SC Yang J Bourcier K Bradshaw EM Seyfert-Margolis V Nepom GT Kwok WW Hafler DA Increased Frequencies of Myelin Oligodendrocyte Glycoprotein/MHC Class II-Binding CD4 Cells in Patients with Multiple Sclerosis J Immunol 2011 187 1039 1046 10.4049/jimmunol.1001543 21653833 PMC3131477

[B21] Mantegazza R Cristaldini P Bernasconi P Baggi F Pedotti R Piccini I Mascoli N Mantia LL Antozzi C Simoncini O Cornelio F Milanese C Anti-MOG autoantibodies in Italian multiple sclerosis patients: specificity, sensitivity and clinical association Int Immunol 2004 16 559 565 10.1093/intimm/dxh056 15039386

[B22] Achenbach P Ziegler AG Diabetes-related antibodies in euglycemic subjects Best Pract Res Clin Endocrinol Metab 2005 19 101 117 10.1016/j.beem.2004.11.009 15826925

[B23] Zhou D Srivastava R Nessler S Grummel V Sommer N Bruck W Hartung HP Stadelmann C Hemmer B Identification of a pathogenic antibody response to native myelin oligodendrocyte glycoprotein in multiple sclerosis Proc Natl Acad Sci USA 2006 103 19057 19062 10.1073/pnas.0607242103 17142321 PMC1748176

[B24] Burrer R Buchmeier MJ Wolfe T Ting JP Feuer R Iglesias A von Herrath MG Exacerbated pathology of viral encephalitis in mice with central nervous system-specific autoantibodies Am J Pathol 2007 170 557 566 10.2353/ajpath.2007.060893 17255324 PMC1851853

[B25] Antel JP Bar-Or A Do myelin-directed antibodies predict multiple sclerosis? N Engl J Med 2003 349 107 109 10.1056/NEJMp030098 12853581

