# Supplementary material for: Conformational epitopes of myelin oligodendrocyte glycoprotein are targets of potentially pathogenic antibody responses in multiple sclerosis
Source: J Neuroinflammation. 2011 Nov 17;8:161. doi: 10.1186/1742-2094-8-161 (PMC3238300; doi:10.1186/1742-2094-8-161)
Supplement: Additonal file 1 — SDS-PAGE and CD-spectroscopy of MOG. Figures of a SDS-PAGE demonstrating high purity of the three recombinant MOG isoforms, and of a circular dichroism spectroscopy experiment proving correct β-sheet folding. [file 1742-2094-8-161-S1.PDF]

<u>Additional file 1</u>

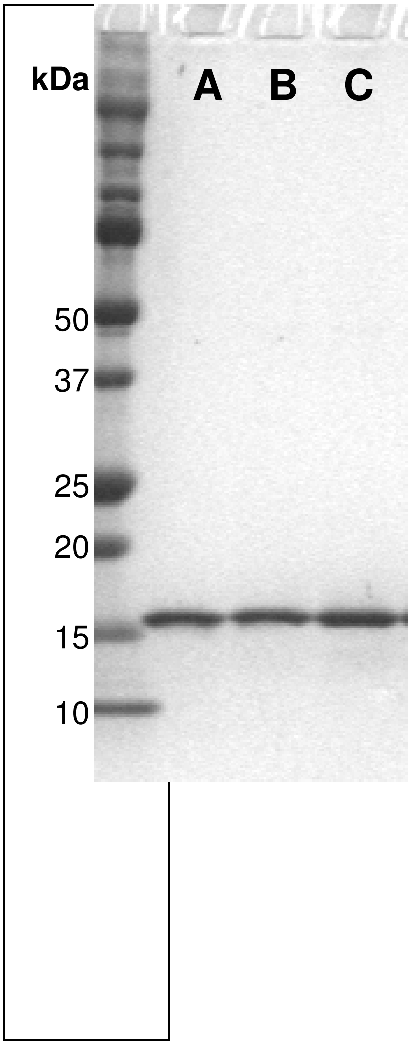

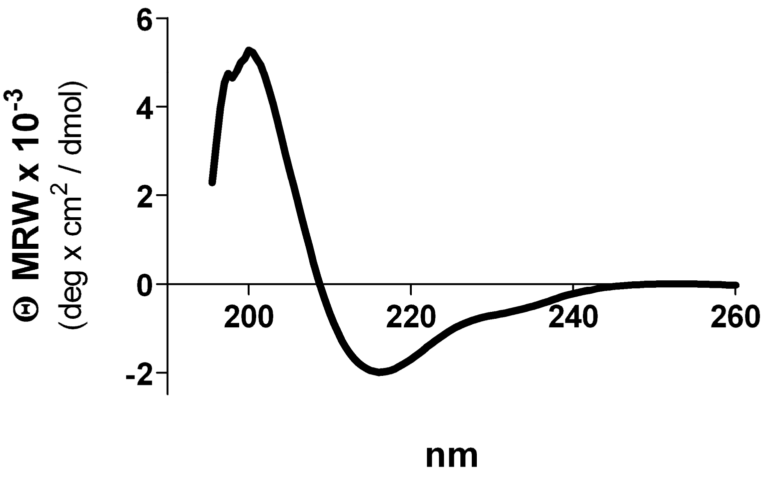

<u>Additional file 1:</u> **SDS-PAGE and CD-spectroscopy of MOG**

<u>Left:</u> SDS-PAGE of 2.0 ug per lane of ratMOG$_{125}$ (A), rhMOG$_{125}$ (B), rhMOG$_{118}$ (C), respectively. Samples were separated according to manufacturer's protocol over a 10-20 % TRIS-HCl gel (Ready Gel, Bio-RAD, Hercules, CA) and stained with Coomassie (Biosafe Coomassie, Bio-RAD). Note that all proteins appear at approximately 15 kDa, do not form dimers and are highly pure (>95 %). Left lane: broad-base molecular weight marker (Precision Plus, Bio-RAD).

Right: circular dichromism (CD) spectroscopy, exemplified for $rhMOG_{118}$. CD spectra were recorded with a Jasco spectropolarimeter at 25℃ at the biophysical research core facility. The cell path length was 10 mm; $rhMOG_{118}$ was analyzed at 0.55 mg/ml in PBS. CD results were the mean of four spectra, reported as mean residue weight ellipticity (θ MRW).
Characteristic for a β-sheet folding, the far-UV CD spectra include a minima at 216 nm and a positive peak near 200 nm [11,12].
