# Supplementary material for: Conformational epitopes of myelin oligodendrocyte glycoprotein are targets of potentially pathogenic antibody responses in multiple sclerosis
Source: J Neuroinflammation. 2011 Nov 17;8:161. doi: 10.1186/1742-2094-8-161 (PMC3238300; doi:10.1186/1742-2094-8-161)
Supplement: Additonal file 2 — Clinical characteristics of healthy control, CIS and MS samples tested. Table compiling the demographic and clinical features of the three patient groups. [file 1742-2094-8-161-S2.PDF]

Additional file 2: **Clinical characteristics of healthy control, CIS and MS samples tested**

	N	Sex (M/F)	Age (years)		Disease duration (months)		EDSS	
			Median	(IQR)	median	(IQR)	median	(IQR)
**HC**	164	61/103	42 [b]	(29-56)				
**CIS**	69	23/46	29	(25-36)			2.0	(1.5-3.0)
**MS**	325	119/206	45	(37-52)	76	(19-135)	3.0	(1.5-5.0)
**RR-MS**	192	57/135 [a]	40 [c]	(33-49)	45	(12-94)	2.0 [c]	(1.0-3.0)
**SP-MS**	69	30/39	48	(42-53)	150 [d]	(108-214)	6.0	(4.0-6.5)
**PP-MS**	64	32/32	52	(45-58)	82 [e]	(24-144)	4.0	(3.5-6.0)

IQR – interquartile range; CIS – clinically isolated syndrome

[a] $p<0.05$ vs. PP-MS (χ^2 test with Yates' continuity correction)

[b] $p<0.001$ vs. PP-MS (Kruskal-Wallis test with Dunn's posthoc test)

[c] $p<0.001$ vs. SP-MS and PP-MS (Kruskal-Wallis test with Dunn's posthoc test)

[d] $p<0.001$ vs. RR-MS and PP-MS (Kruskal-Wallis test with Dunn's posthoc test)

[e] $p<0.05$ vs. RR-MS (Kruskal-Wallis test with Dunn's posthoc test)
