# Supplementary material for: Conformational epitopes of myelin oligodendrocyte glycoprotein are targets of potentially pathogenic antibody responses in multiple sclerosis
Source: J Neuroinflammation. 2011 Nov 17;8:161. doi: 10.1186/1742-2094-8-161 (PMC3238300; doi:10.1186/1742-2094-8-161)
Supplement: Additonal file 3 — Dilution series of samples with high titers. Figure of an ELISA of two-fold serial serum dilutions against the three MOG isoforms to demonstrate sustained ELISA reactivity of samples with high IgG concentrations (> 95th percentile) beyond dilutions of 1/2,000. [file 1742-2094-8-161-S3.PDF]

Additional file 3

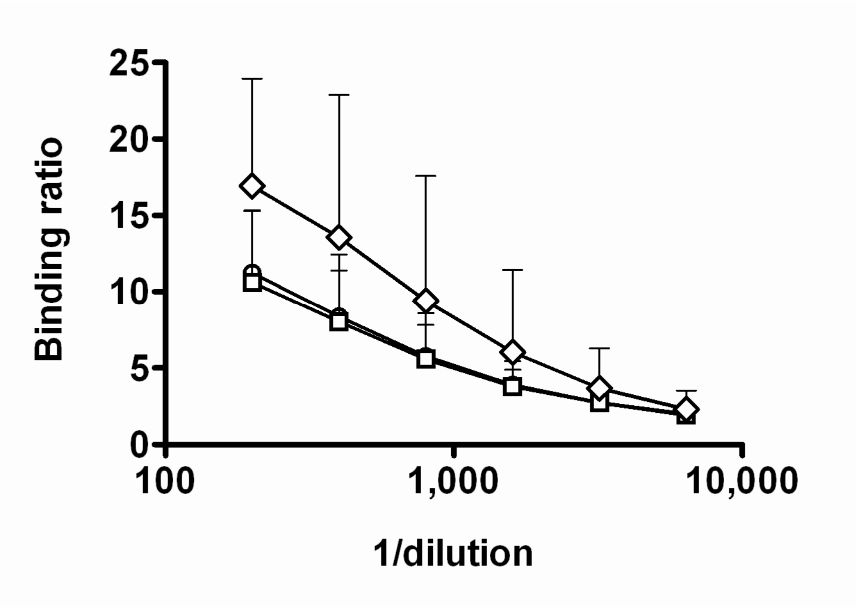

Additional file 3: Dilution series of samples with high titers

Two-fold serial serum dilutions starting at 1/200. Results are expressed as binding ratios (OD_{MOG} / OD_{BSA}); error bars represent SD. Samples with high IgG concentrations (>95th percentile) can be diluted beyond dilutions of 1/2,000 retaining ELISA reactivity against $rhMOG_{118}$ (-○-), $rhMOG_{125}$ (-□-) and $ratMOG_{125}$ (-◊-).
