# Supplementary material for: Conformational epitopes of myelin oligodendrocyte glycoprotein are targets of potentially pathogenic antibody responses in multiple sclerosis
Source: J Neuroinflammation. 2011 Nov 17;8:161. doi: 10.1186/1742-2094-8-161 (PMC3238300; doi:10.1186/1742-2094-8-161)
Supplement: Additonal file 4 — Persistent anti-MOG reactivity in serial samples. Figure of an ELISA reactivity against rhMOG118 of six samples, for which longitudinal samples were drawn every three months over 18 months; the data prove that anti-MOG IgG concentrations vary only within the limits of the assay. [file 1742-2094-8-161-S4.PDF]

Additional file 4

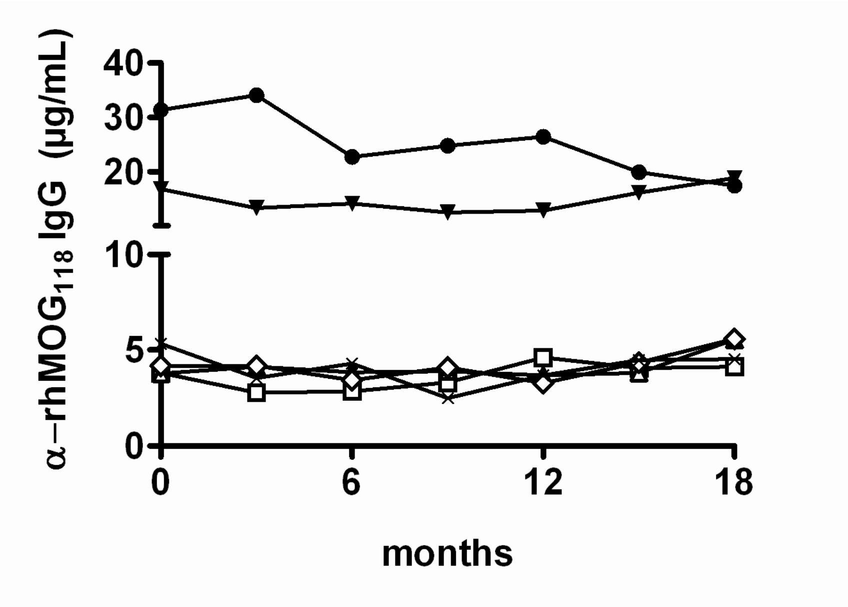

Additional file 4: **Persistent anti-MOG reactivity in serial samples**

Anti-rhMOG$_{118}$ reactivity of six RR-MS, two of which were identified with high-titer anti-MOG reactivity, for whom longitudinal samples were drawn every 3 months over 18 months. -●- and -▼- denote samples with high-titer reactivity vs. -□-, -◊-, -Δ- and –X-. Results are expressed as specific IgG concentrations (µg/mL). The magnitudes of reactivity vary within the limits of the assay, but high-titer samples remain high and vice versa, i.e. the two samples identified as high-titer responders did not loose their high reactivity, while none of the four other samples showed increased titers over time.
